# Proderm technology: a water- based lipid delivery system for dermatitis that penetrates viable epidermis and has antibacterial effects

**DOI:** 10.1186/s12895-019-0082-8

**Published:** 2019-01-22

**Authors:** Alexandra Charruyer, Mats Silvander, Melinda Caputo-Janhager, Isabelle Raymond, Ruby Ghadially

**Affiliations:** 1 Department of Dermatology, University of California San Francisco, San Francisco, California USA; 2 Department of Veterans Affairs San Francisco, San Francisco, USA; 3 Aerosol Scandinavia AB Vallentuna, Vallentuna, Sweden; 4 Exeltis, Florham Park, USA; 5 Epithelial Section of the UCSF Eli and Edythe Broad, Center of Regeneration Medicine and Stem Cell Research, 1700 Owens Street, Room 324, San Francisco, CA94158 USA

**Keywords:** Skin barrier, Epidermis, Penetration, Fatty acids, Antimicrobial, *Staphylococcus aureus*, *Escherichia coli*, Dermatitis, Physiological lipids

## Abstract

**Background:**

A defective skin barrier and bacterial colonization are two important factors in maintenance and progression of atopic dermatitis and chronic allergic/irritant hand dermatitis. A water-based lipid delivery system containing physiologic lipids was previously shown to be a useful adjunct in the treatment of hand dermatitis. We tested the ability of this formulation to penetrate into the viable epidermis and in addition assessed its antibacterial properties.

**Methods:**

Epidermal penetration of the product was assessed by fluorescence microscopy. Recovery of *Escherichia coli* and *Staphylococcus aureus* MRSA from skin treated with Neosalus® foam was quantified.

**Results:**

Components of Neosalus® penetrated the stratum corneum and were distributed throughout the viable epidermis. Neosalus® significantly decreased recovery of both *Staphylococcus aureus* and *Escherichia coli* from the skin surface.

**Conclusions:**

The ability of components of Neosalus® to be taken up into the viable epidermis and potentially made available for incorporation into the barrier lipids, combined with antibacterial properties, indicate that this formulation may be valuable not only in chronic hand dermatitis, but also in various other forms of dermatitis.

**Trial registration:**

Current Controlled Trials ISRCTN18191379, 28/12/2018, retrospectively registered.

## Background

Research continues to reaffirm the important role of the epidermal barrier in maintaining cutaneous health and implicates barrier dysfunction in the pathogenesis of contact dermatitis, ichthyosis, psoriasis, atopic dermatitis [1], and in allergic and irritant contact dermatitis [2, 3]. Barrier dysfunction is linked to increased transepidermal water loss and decreased hydration in pathological dry skin [4] . Barrier dysfunction facilitates secondary infection by bacteria, viruses, and fungi [5]. *Staphylococcus aureus* is increasingly implicated as a factor in the pathogenesis of atopic dermatitis [6]. The pooled prevalence of *Staphylococcus aureus* colonization of atopic eczema patients was 70% for lesional skin and both lesional and non-lesional skin are commonly colonized [7–9]. Agents that combat the barrier dysfunction and bacterial colonization have potential therapeutic value.

The skin barrier resides principally in the stratum corneum and is comprised of ceramides, cholesterol, and free fatty acids. The skin barrier prevents the loss of water and the penetration of foreign substances, both pathogenic and therapeutic. The ability of substances to cross the skin barrier depends not only on the properties of the substance but also on the condition of the skin [10]. Proderm Technology**™** is a water-lipid-based foam delivery system that was developed to supply active ingredients to the skin without disrupting the natural skin barrier. Its unique properties are believed to be derived from its well-defined mixture of fatty acids in free and physically bound forms and 80% water. The lipid components of Neosalus® include medium-chain free fatty acids, that occur naturally in the skin. Proderm technology confers delivery properties that are expected to support skin barrier repair better than purely fat- or water-based formulations. Other ingredients in Neosalus® include glycerin and dimethicone. Glycerin is both humectant, increasing moisture in the stratum corneum by attracting water from the dermis and the environment, and emollient, affecting epidermal biomechanics by increasing hydration [11]. Unmodified silicones, such as dimethicone, remain on or near the skin surface, as their molecules are too large to penetrate the epidermis, resulting in a barrier to moisture loss and protection against allergens and irritants [11].

**Fig. 1:**
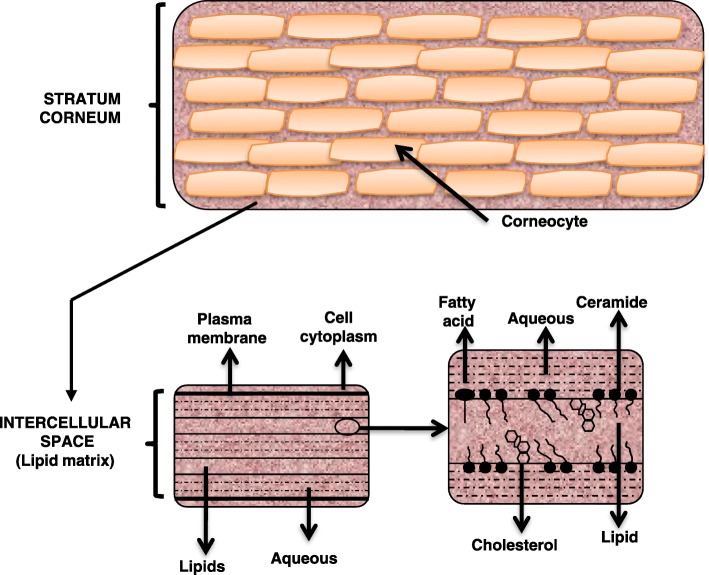
Hydrophobic and hydrophilic regions of the intercellular domains: The water-lipid nature of Neosalus™ is expected to support skin barrier repair better than purely fat- or water-based formulations. Adapted from Escobar-Chavez et al. 2012 [13]

The topical delivery of therapeutic agents into the epidermis requires sufficient penetration of the stratum corneum to the viable epidermis below. Accordingly, “enhancers” are sometimes used to optimize penetration. However, while enhancing penetration, these agents tend to disrupt the cutaneous barrier and may cause irritation and increase skin sensitivity. Thus, the ideal formulation for topical drug delivery should be one that is capable of solubilizing both hydrophilic and lipophilic substances and enhances uptake without damaging the skin or the skin barrier [12]. Permeability is governed by hydrophobic and hydrophilic channels (Fig. 1, [13]). The majority of skin surface lipids are segregated into crystalline/gel domains bordered by “grain borders” where lipids exist in a fluid, crystalline state. The fluid character of this area permits the diffusion of lipid and hydrophobic molecules through the system on downhill gradients [12]. If the lipid content decreases, the “gate keeper” function of the barrier becomes compromised and water loss increases, leading to dry irritated skin. The Proderm water-lipid-based foam exhibits a distribution of water and free fatty acids; presents in anionic (hydrophilic) and neutral (hydrophobic) form (U.S. Patent 5,993,830). Previous studies have shown the benefit of topical skin protectants using Proderm technology to diminish occupational and home exposure to irritants and allergens [14, 15]**.** The studies here were performed to test the ability of Neosalus® to penetrate into the viable epidermis and to test its antimicrobial properties; properties expected to underlie the therapeutic efficacy seen.

## Methods

### Structural characterization of Neosalus®

Neosalus foam was investigated by means of light microscopy and electron microscopy. Electron micrographs were generated through the cryo-Transmission Electron Microscope Technique in which a thin film of sample is vitrified and transmitted by electrons. The method of cTEM has been described in detail elsewhere [16].

### Penetration of fatty acids associated with Neosalus® into viable epidermis

To measure the penetration of the physiological free fatty acids associated with Neosalus® foam, fluorescent-labeled medium-chain fatty acid 4,4-Difluoro-5-(2-Thienyl)-4-Bora-3a,4a-Diaza-*s*-Indacene-3-Dodecanoic Acid (Bodipy™, Molecular Probes) was added in trace amounts to the foam delivery system to travel with its fatty acid content. Similarly, tracer was added to dimethicone and petrolatum as controls. In previous studies, petrolatum was not observed to penetrate into the viable epidermis [17]. Full-thickness murine skin biopsies (hr mice, The Jackson Laboratory) were taken 2 h after application of the test substances (mice were not scarified), as in previous similar studies from others [18]. Samples were counterstained with a nuclear dye (DAPI, blue) and fluorescence microscopy (Axio Plan Z, Carl Zeiss Inc., Thornwood, NY) was used to determine the location of the test substances 2 h after application. These studies were performed with approval from and following the guidelines of our Institutional animal care and use facility (IACUC).

### Antimicrobial effects of Neosalus®

Institutional Review Board approval was obtained for this study and appropriate informed consent was obtained from all research participants (Gallatin Institutional Review Board-Approved Protocol #090426–150). Twenty healthy subjects were evaluated to determine the antimicrobial efficacy of the compound formulation. Recoveries of *Escherichia coli* (ATCC #11229) and *Staphylococcus aureus* MRSA (ATCC #33593) populations from the skin of the treated forearms of ten subjects per bacterial species were compared to recoveries from the skin of their untreated (control) forearms. After each subject had completed a 7-day restriction period (no solvents, detergents, tanning, or swimming) 1 ml of the microbial suspension was applied to the skin of a randomly assigned forearm of each subject. The subject’s contralateral forearm served as an untreated control, receiving no treatment of any kind. Four sites were delineated on the skin of each forearm, and 10 min following product application, the sites were exposed to the randomly assigned challenge suspension for contact times of 5 min, 10 min, 20 min, and 40 min. Microbial suspensions were recovered from each site by cylinder sampling and then skin sites decontaminated with 70% IPA.

Duplicate spiral plates or duplicate spread plates were prepared from cylinder samples (I0° dilution) on MacConkey Agar (MAC) for *Escherichia coli* (ATCC #11229) and Hardy Chrom Staph aureus (CHROM) for *Staphylococcus aureus aureus* (ATCC #33593). These were incubated at 30° ± 2 °C for 48 h and at 35° ± 2 °C for 24 h, respectively, or until sufficient growth was observed. Colonies were counted and data recorded using the Q-Count plate counting system or equivalent.

To prepare the microbial suspensions, sterile tubes of Tryptic Soy Broth (TSB) were inoculated from cryogenic stock cultures or lyophilized vials containing *Escherichia coli* (ATCC # ll229) and *Staphylococcus aureus* MRSA {ATCC #33593). Cultures were incubated at 30° ± 2 °C for 24 h. Immediately prior to the procedure, *cultures* were transferred from Tryptic Soy Agar to Phosphate Buffer Solution (PBS) and diluted to 1.0 × 10^9^ CFU/mL.

## Results

### Neosalus® water-lipid-based foam penetrates into the epidermis

**Fig. 2:**
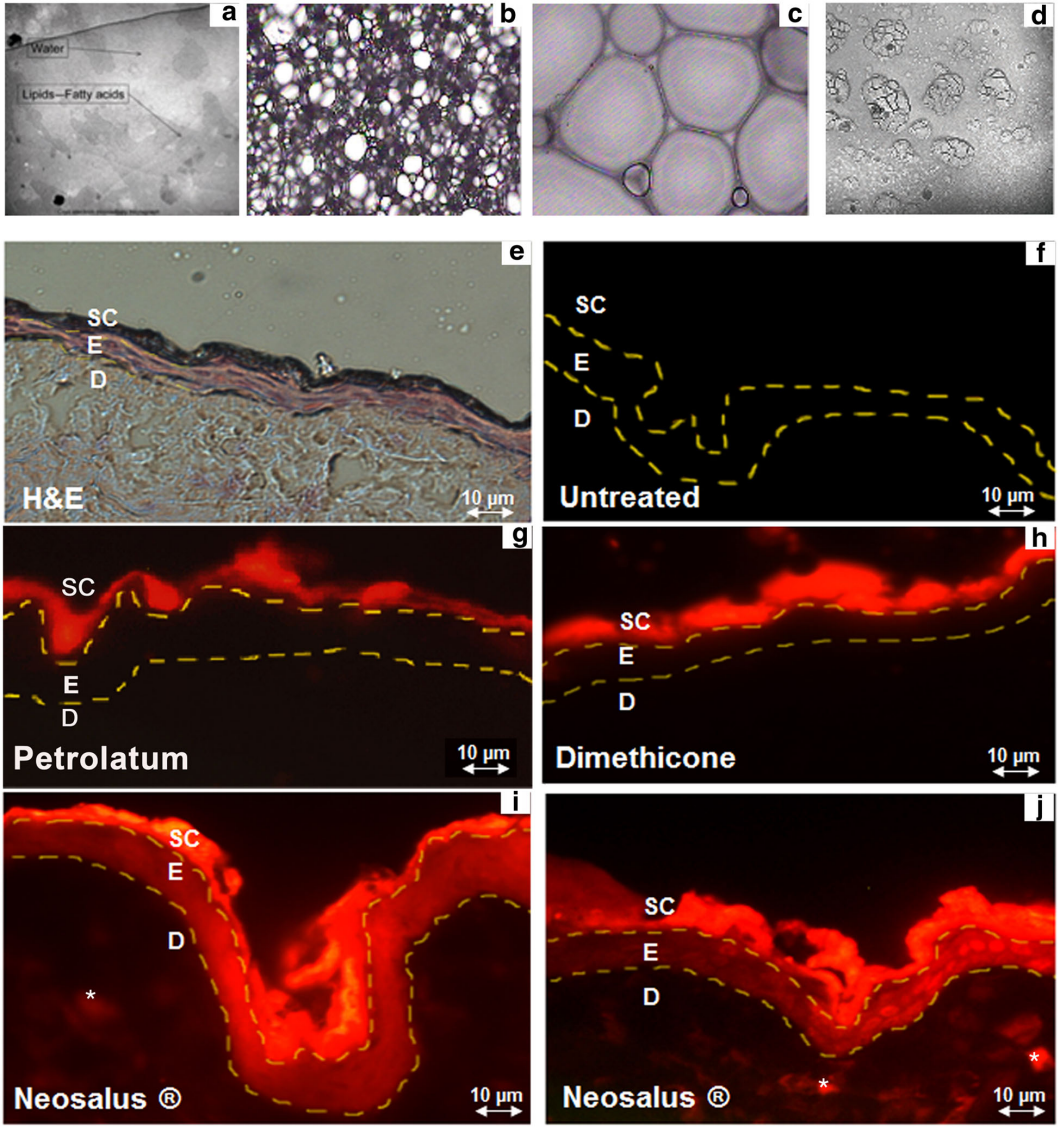
Visualization of Neosalus and its penetration into epidermis. **a** The foam exhibits a distribution of water and free fatty acids; which are present in anionic (hydrophilic) and neutral (hydrophobic) form. **b**, **c** Light micrographs of foam showing the structure of the foam with its liquid layers between gas (propellant) cavities. **d** Structures within foam are less than 1 μm (cTEM). **e** Normal untreated skin (H&E). **f** Untreated normal skin shows minimal fluorescence. **g** Fluorescence seen in the stratum corneum of skin treated with fluorescent-labeled petrolatum. **h** Fluorescence seen in the stratum corneum of skin treated with fluorescent-labeled dimethicone. **i**, **j** Skin treated with fluorescent-labeled Neosalus® shows fluorescence throughout the stratum corneum and epidermis. SC: stratum corneum, E: epidermis, D: dermis, * autofluorescence of collagen fibers

The Proderm water-lipid-based foam exhibits a distribution of water and free fatty acids; which are present in anionic (hydrophilic) and neutral (hydrophobic) form (Fig. 2a). The foam consists of gas cavities surrounded by lipid lamellae (Fig. 2b,c). Within the lamellae, structures are present that are smaller than a micrometer (Fig. 2d). It was hypothesized that physiological lipids in Neosalus® would penetrate into the viable epidermis while dimethicone and petrolatum would remain on the skin surface as a protective barrier. To measure the penetration of physiological lipids in Neosalus® foam, fluorescent-labeled fatty acid (Bodipy, Molecular Probes) was added in trace amounts to the foam delivery system to mark its fatty acid content. The tracer was also added to dimethicone and petrolatum as controls. The foam, dimethicone, and petrolatum, were applied to the skin and biopsy samples taken 2 h after application. Fluorescence microscopy was used to determine the degree of penetration and location of the test substances. Fluorescence microscopy of untreated normal skin showed minimal fluorescence (Fig. 2e, f). While petrolatum (Fig. 2g) and dimethicone (Fig. 2h) showed strong fluorescence only in the stratum corneum, components of Neosalus® penetrated the stratum corneum and were distributed throughout the viable epidermis, as demonstrated by bright fluorescence (Fig. 2i, j). Thus the fluorescent free fatty acids in Neosalus® were delivered to the viable epidermis. These lipids, as previously shown, are available as a source of lipids for de novo barrier formation [18].

### Antimicrobial properties of Neosalus

**Fig. 3:**
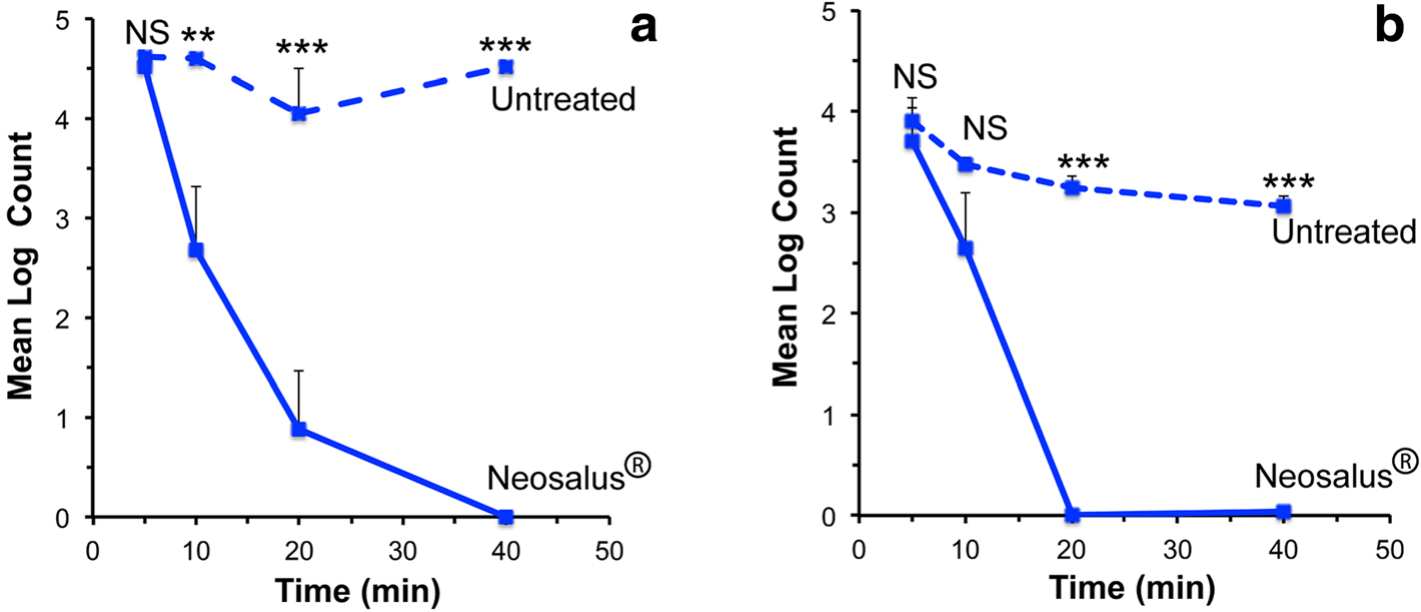
Antibacterial properties of Neosalus®. **a** Mean Log_10_ Colony Counts of *Staphylococcus aureus* recovered from skin treated with Neosalus® or untreated skin. **b** Mean Log_10_ Colony Counts of *Escherichia coli* recovered from skin treated with Neosalus® or untreated skin

A clinical study was performed to evaluate the antimicrobial properties of Neosalus® (Fig. 3, Tables 1 and 2). Twenty patients underwent application of Neosalus to one forearm while the other forearm was used as an untreated control. Ten minutes after product application, the sites were exposed to either *Staphylococcus aureus* or *Escherichia coli*, for contact times of 5, 10, 20, and 40 min, and then skin surface bacteria collected and cultured. The Neosalus® treated forearm showed significantly decreased recovery of *Staphylococcus aureus* at 10 min, with eradication of bacteria after 40 min (Fig. 3a). The Neosalus® treated forearm showed eradication of *Escherichia coli* at 20 min (Fig. 3b). Thus the product showed evidence of potential antibacterial properties.

**Table 1:** Mean Log_10_ Colony Counts of *Staphylococcus aureus* recovered from treated vs. untreated skin

Treatment	Sample Time	Sample Size	Mean	Standard Deviation	95% Confidence Interval	*p* value
Test Product	5 Minutes	10	4.52	0.17	4.39 to 4.64	0.172
Untreated		10	4.61	0.12	4.53 to 4.70	
Test Product	10 Minutes	10	2.68	2.00	1.25 to 4.11	0.014
Untreated		10	4.60	0.11	4.52 to 4.68	
Test Product	20 Minutes	10	0.89	1.83	−0.42 to 2.20	0.000
Untreated		10	4.05	1.43	3.03 to 5.08	
Test Product	40 Minutes	10	0.00	0.00	0.00 to 0.00	0.000
Untreated		10	4.52	0.16	4.40 to 4.63

**Table 2:** Mean Log_10_ Colony Counts of *Escherichia coli* recovered from treated vs. untreated skin

Treatment	Sample Time	Sample Size	Mean	Standard Deviation	95% Confidence Interval	*p* value
Test Product	5 Minutes	10	3.70	1.36	2.73 to 4.67	0.648
Untreated		10	3.91	0.41	3.62 to 4.20	
Test Product	10 Minutes	10	2.65	1.71	1.43 to 3.87	0.166
Untreated		10	3.47	0.24	3.30 to 3.65	
Test Product	20 Minutes	10	0.00	0.00	0.00 to 0.00	0.000
Untreated		10	3.25	0.35	3.00 to 3.50	
Test Product	40 Minutes	10	0.03	0.11	−0.04 to 0.11	0.000
Untreated		10	3.06	0.32	2.83 to 3.29	

## Discussion

Frustration with therapy for dermatitis is common among both treating physicians and affected patients. Numerous therapies are effective for acute management of dermatitis, quell inflammation, and improve symptoms. Frustration grows as patients taper and withdraw topical corticosteroids and dermatitis recurs. In recent years “barrier-repair” creams, topical skin protectants, and other moisturizing formulations intended to forestall dermatitis recurrence have helped prolong clearance while decreasing patients’ exposure to topical corticosteroids. Understanding the mechanisms by which various agents aid in barrier repair, decrease colonization, and are anti-inflammatory will allow us to continue to improve formulations that can provide key therapeutic adjuncts in the treatment of dermatitis.

Previous studies have shown the benefit of topical skin protectants using Proderm technology [17, 18]. Underlying the therapeutic efficacy, here we show that components of the water-lipid-based delivery system, Neosalus®, can effectively penetrate into the viable epidermis. The free fatty acids in Proderm could penetrate the stratum corneum and be distributed throughout the viable epidermis.

While many components of Neosalus could contribute to antibacterial properties, long chain fatty acids are known to be bactericidal or/and inhibit bacterial growth [19–21]. Also, additionally or alternatively, barrier repair could be an indirect mechanism for antibacterial properties. Antibacterial properties would constitute an exciting benefit for patients with atopic dermatitis who are mostly colonized by *Staphylococcus aureus*. Importantly, a limitation of this work is that long-term benefits to skin microflora have not yet been investigated and this will be important.

Topical steroids are commonly used for the treatment of dermatitis, but they are associated with many side effects, including skin atrophy. An effective barrier-repair and protection foam is important for acute and chronic treatment of dermatitis and for some may be useful as an alternative to topical steroids and immunomodulators. Furthermore, barrier maintenance properties and bactericidal properties may help prevent flares of dermatitis.

## Conclusions

Constituents of Neosalus® are taken up into the viable epidermis making them available for lipid incorporation into the barrier. These studies also indicate that Neosalus® has possible antibacterial properties. These properties suggest that this formulation may be valuable not only in chronic hand dermatitis, but also in various other forms of dermatitis.
